# Decellularized Cartilage May Be a Chondroinductive Material for Osteochondral Tissue Engineering

**DOI:** 10.1371/journal.pone.0121966

**Published:** 2015-05-12

**Authors:** Amanda J. Sutherland, Emily C. Beck, S. Connor Dennis, Gabriel L. Converse, Richard A. Hopkins, Cory J. Berkland, Michael S. Detamore

**Affiliations:** 1 University of Kansas Medical Center, Kansas City, Kansas, United States of America; 2 Bioengineering Graduate Program, University of Kansas, Lawrence, Kansas, United States of America; 3 Orbis Biosciences, Kansas City, Kansas, United States of America; 4 Children’s Mercy Hospital, Cardiac Surgery Research Lab, Ward Family Center for Congenital Heart Disease, Kansas City, Missouri, United States of America; 5 Department of Chemical and Petroleum Engineering, University of Kansas, Lawrence, Kansas, United States of America; 6 Department of Pharmaceutical Chemistry, University of Kansas, Lawrence, Kansas, United States of America; University of Pittsburgh, UNITED STATES

## Abstract

Extracellular matrix (ECM)-based materials are attractive for regenerative medicine in their ability to potentially aid in stem cell recruitment, infiltration, and differentiation without added biological factors. In musculoskeletal tissue engineering, demineralized bone matrix is widely used, but recently cartilage matrix has been attracting attention as a potentially chondroinductive material. The aim of this study was thus to establish a chemical decellularization method for use with articular cartilage to quantify removal of cells and analyze the cartilage biochemical content at various stages during the decellularization process, which included a physically devitalization step. To study the cellular response to the cartilage matrix, rat bone marrow-derived mesenchymal stem cells (rBMSCs) were cultured in cell pellets containing cells only (control), chondrogenic differentiation medium (TGF-β), chemically decellularized cartilage particles (DCC), or physically devitalized cartilage particles (DVC). The chemical decellularization process removed the vast majority of DNA and about half of the glycosaminoglycans (GAG) within the matrix, but had no significant effect on the amount of hydroxyproline. Most notably, the DCC group significantly outperformed TGF-β in chondroinduction of rBMSCs, with collagen II gene expression an order of magnitude or more higher. While DVC did not exhibit a chondrogenic response to the extent that DCC did, DVC had a greater down regulation of collagen I, collagen X and Runx2. A new protocol has been introduced for cartilage devitalization and decellularization in the current study, with evidence of chondroinductivity. Such bioactivity along with providing the ‘raw material’ building blocks of regenerating cartilage may suggest a promising role for DCC in biomaterials that rely on recruiting endogenous cell recruitment and differentiation for cartilage regeneration.

## Introduction

Degeneration of articular cartilage can be caused by traumatic injury or arthritis. Articular cartilage regeneration is a particularly difficult problem because cartilage has a limited capacity for self-repair and low vascularity [1, 2]. Current clinical treatments for cartilage degeneration include autologous chondrocyte implantation (ACI), mosaicplasty, microfracture, and allograft implants. These treatments have limited success in fully regenerating functional articular cartilage. The repair tissue is often fibrous with inferior mechanical performance compared to native cartilage.

In the past, tissue engineering has aimed to regenerate articular cartilage utilizing synthetic biomaterials due to the ability to alter and control synthetic materials’ mechanical properties [3–5]. These synthetic materials, however, have limited ability to recruit and differentiate stem cells without added biological components such as peptide sequences or growth factors. Recently, acellular extracellular matrix (ECM) materials have become popular because the matrices retain the native structure of cartilage, which provides cells with both mechanical and chemical signals to aid in stem cell differentiation and recruitment, and ultimately in tissue regeneration [6–8]. ECM materials can induce differentiation and regeneration without additional biologic additives, which may be an attractive alternative from both cost and regulatory standpoints [9].

ECM materials can be obtained from either cell-derived matrices secreted during *in vitro* culture (CDM) or from native tissue [6, 10–14]. Both types of matrices have been either decellularized to fully remove all cellular components and nucleic acids or devitalized to kill all remaining cells within the matrix without completely removing them. In contrast, fully decellularized native cartilage (DCC) tissue presents a unique challenge because the dense ECM makes full decellularization difficult due to diffusion limitations [13, 15]. The tissue is often mechanically disrupted to increase the efficacy of chemical decellularization but destroys the mechanical properties of the matrix [14, 16, 17]. The dense nature of native articular cartilage also restricts cell migration into the matrix [13]. Successful decellularization results in an acellular matrix that has low immunogenicity with the same biochemical make-up as native cartilage [13, 18, 19]. Devitalized cartilage (DVC), on the other hand, may still contain antigenic cell surface markers. Both types of cartilage matrix can additionally be combined with synthetic biomaterials or crosslinked to achieve the desired mechanical properties or shape [20, 21].

Previous studies have investigated the use of DCC and DVC as chondroinductive materials, but have not fully characterized the materials through the decellularization processes [11, 17, 19, 21, 22]. Other studies have also chemically or physically crosslinked the DCC or incorporated the DCC into synthetic material scaffolds prior to *in vitro* cell culture. The cellular response to native, non-crosslinked DCC has not been investigated. Additionally, DCC has not been directly compared to devitalized native cartilage (DVC) *in vitro*, which is the first step in determining which matrix components are crucial for chondrogenesis.

The primary goal of the current study was to assess changes to native cartilage matrix throughout both decellularization and devitalization protocols and to further investigate the chondroinductive potential of cartilage ECM materials. The current study investigated the combined physical and chemical decellularization and the physical devitalization of native porcine cartilage processing effects on the biochemical and DNA content of the material at different steps of the decellularization process. The chondroinductive nature of non-crosslinked DCC were also examined for future use in cartilage tissue engineering by assessing the differentiation of rat bone marrow derived mesenchymal stem cells (rBMSCs) in 3D pellet culture without added growth factors. Future applications of DCC use include a limitless variety of 3D scaffolding systems, from pastes to hydrogels to solid scaffolds such as fibers, microspheres or 3D-printed shapes.

## Materials and Methods

### Tissue Retrieval, Decellularization, and Devitalization

**Fig 1:**
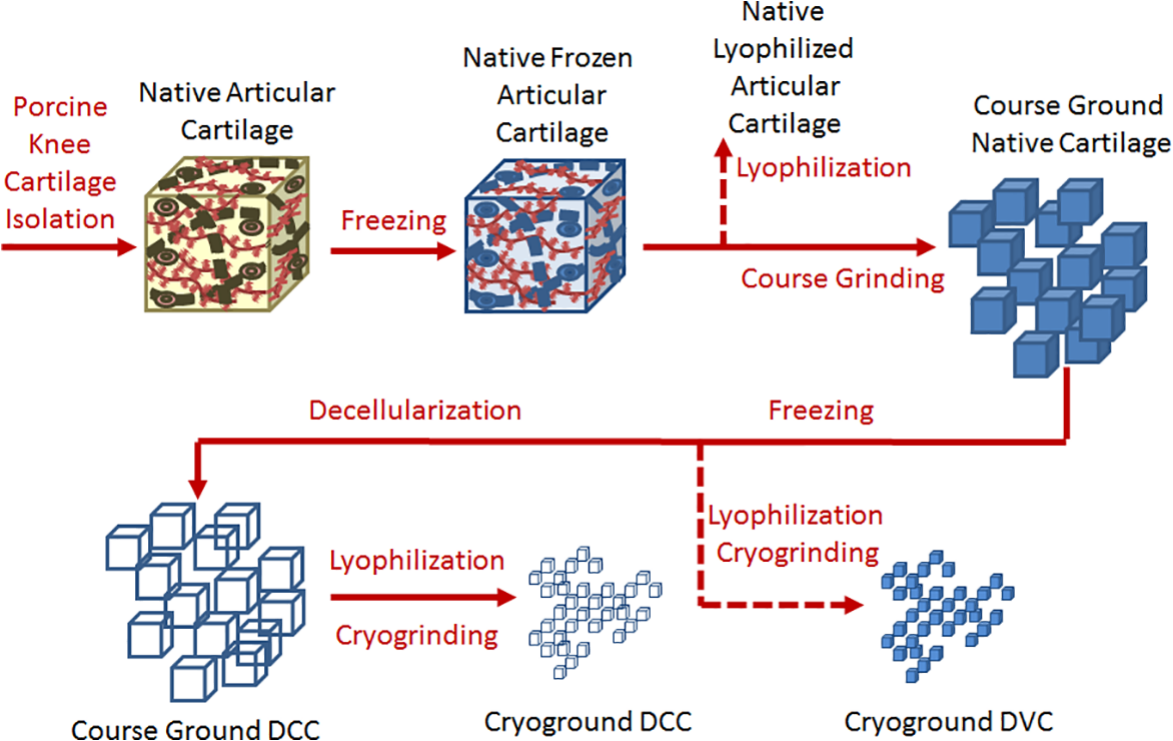
Schematic of Porcine Cartilage Processing. Red lines and text indicate the order and type of processing. Red dashed lines indicate locations in the process where deviations in processing were made to provide controls.

Ten porcine knee and hip joints were purchased from a local abattoir following sacrifice (120 kg, mixed breed, mixed gender) (Bichelmeyer Meats, Kansas City, KS) and processed according to the schematic in Fig 1. Articular cartilage from both the knee and hip joints was carefully removed and collected using scalpels. The cartilage was rinsed in phosphate buffered saline (PBS) and stored at -20°C. Following freezing, the cartilage was coarsely ground using a cryogenic tissue grinder (BioSpec Products, Bartlesville, OK). The coarsely ground tissue was packaged into dialysis tubing (3500 MWCO) packets for decellularization.

Cartilage was devitalized (i.e., forming DVC) following tissue harvest by immediately freezing at -20°C and then lyophililzing the tissue. The lyophilized tissue was then processed in a freezer-mill and frozen again at -20°C.

The cartilage was decellularized (i.e., forming DCC) using an adapted version of our previously established method using reciprocating osmotic shock, detergent, and enzymatic washes [23]. Reagents were purchased from Sigma-Aldrich (St. Louis, MO) unless otherwise noted. All steps of decellularization were carried out under agitation (200 rpm) at 21°C unless otherwise noted. First, the cartilage packets were placed in hypertonic salt solution (HSS) overnight to disrupt membranes and lyse the cells. Following HSS treatment, the tissue was subjected to 2 cycles of reciprocating triton-X 100 (0.05% v/v) and HSS treatments to further breakdown cellular membranes. The tissue was then treated with benzonase (0.0625 KU ml^-1^) overnight at 37°C to fragment nucleic acids. Sodium-lauroyl sarcosine (NLS, 1% v/v) was then used overnight to further solubilize and remove cells. Next, the tissue was washed with 40% ethanol, followed with organic exchange resins to remove all organic solvents. Lastly, the tissue was removed from the dialysis tubing packages and rinsed with deionized water before freezing.

After decellularization, the tissue was lyophilized for 48 hours and cryo-ground into a fine powder with a freezer-mill (SPEX SamplePrep, Metuchen, NJ).

### Scanning Electron Microscopy

The size and morphology of DCC particles were observed using LEO 1550 field emission scanning electron microscopy (SEM). Prior to imaging, DCC particles were lyophilized and sputter-coated with gold.

### BMSC Harvest and Pellet Formation

Rat bone marrow mesenchymal stem cells (rBMSC) were harvested from the femurs of 4 male Sprague-Dawley rats (200–250 g) following a University of Kansas approved IACUC protocol. The BMSCs were cultured in minimum essential medium (MEM) α culture medium with 10% fetal bovine serum (FBS) and 1% antibiotic-antimycotic (anti-anti) during expansion. At passage 4, the cells were suspended in MEM α culture media at 1x10^6^ cells/mL. 1 mL of cell suspension was added to 25 mg of both DCC and DVC and centrifuged for 5 minutes to form a pellet (n = 5). One million cells were used in each sample to obtain sufficient amounts of RNA for gene expression analysis and 25 mg was chosen empirically as an amount that would be sufficient to have high cell to cartilage matrix contact. The TGF-β and negative control groups contained 10^6^ cells without additional material. The TGF-β control group was cultured in chondrogenic differentiation medium containing 10 ng/mL human recombinant TGF-β_3_ (PeproTech, Rocky Hill, NJ), 50 μg/mL ascorbic acid, 1% Penicillin Streptomycin, 40 μg/mL L-proline, 100 μmol sodium pyruvate, 0.1 μm dexamethasone, 1% insulin-transferrin-selenium 100X (ITS), and 1% non-essential amino acids (NEAA). All other experimental groups, including the negative control group, were cultured in 1 mL of the same MEM α culture media that was used during expansion. The medium was changed every 48 hours.

### Biochemical Analysis

The biochemical content of the cartilage was assessed after each processing step: native hydrated, native frozen, native lyophilized, native cryo-ground (DVC), decellularized coarse ground, and decellularized cryo-ground cartilage (DCC). The DNA content was also assessed at day 1 and 7 of pellet culture (n = 5). Prior to biochemical analysis, all tissue samples were digested in a papain solution for 24–48 hours at 65°C.

Biochemical content was measured as previously reported [24–26]. Briefly, glycosaminoglycan (GAG) content was measured with a dimethylmethylene blue (DMMB) assay kit (Blyscan, Westbury, NY). Total hydroxyproline content was measured using a commercially available hydroxyproline detection kit (Sigma, St. Louis, MO). Double-stranded DNA was detected using a high sensitivity PicoGreen assay kit (Molecular Probes, Eugene, OR). All assay kits were used in accordance with each manufacturer’s guidelines.

### Gene Expression

RNA was isolated and purified from cells using the Qiagen RNeasy mini kit (Valencia, CA). All RNA samples were reverse transcribed using a high capacity cDNA reverse transcription kit (Invitrogen, Carlsbad, CA). Real-time quantitative polymerase chain reaction (qPCR) was performed using a RealPlex MasterCycler (Eppendorf, Hauppauge, NY) and TaqMan gene expression assays using equal concentrations of DNA for each sample. Rat specific Col2A1, Col1A1, Runx2, Sox9, Col10A1, Acan, and GAPDH commercially available primers were used (Invitrogen, Carlsbad, CA). The 2^-ΔΔCt^ method was used to determine the relative expression of each gene with GAPDH used as an endogenous control [27, 28].

### Statistical Analysis

Results are reported as a mean ± standard deviation. SPSS statistical software was used to construct boxplots to remove outliers prior to performing statistical analyses. All statistical analyses were performed using a one way analysis of variance (ANOVA) and Tukey’s *post-hoc* tests. Significance was determined for p<0.05.

## Results

### Tissue Decellularization and Processing

**Fig 2:**
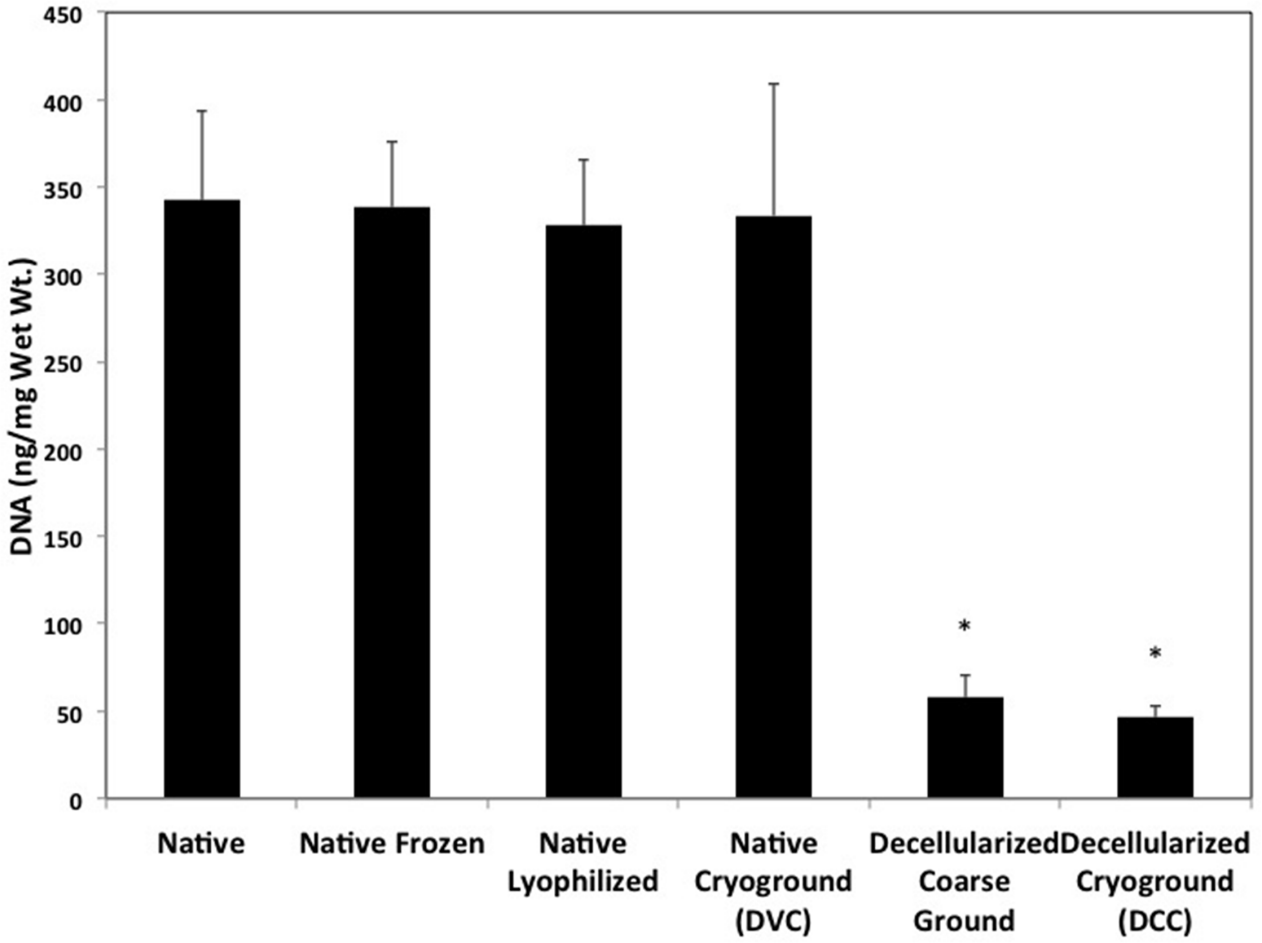
PicoGreen results depicting changes in double stranded (ds)DNA amounts in articular cartilage throughout the decellularization process. Processing the cartilage with both physical and chemical methods significantly reduced the amount of dsDNA in the matrix by 86%. * denotes significance (p<0.01) from native cartilage (n = 6). All results are reported as mean ± standard deviation.

**Fig 3:**
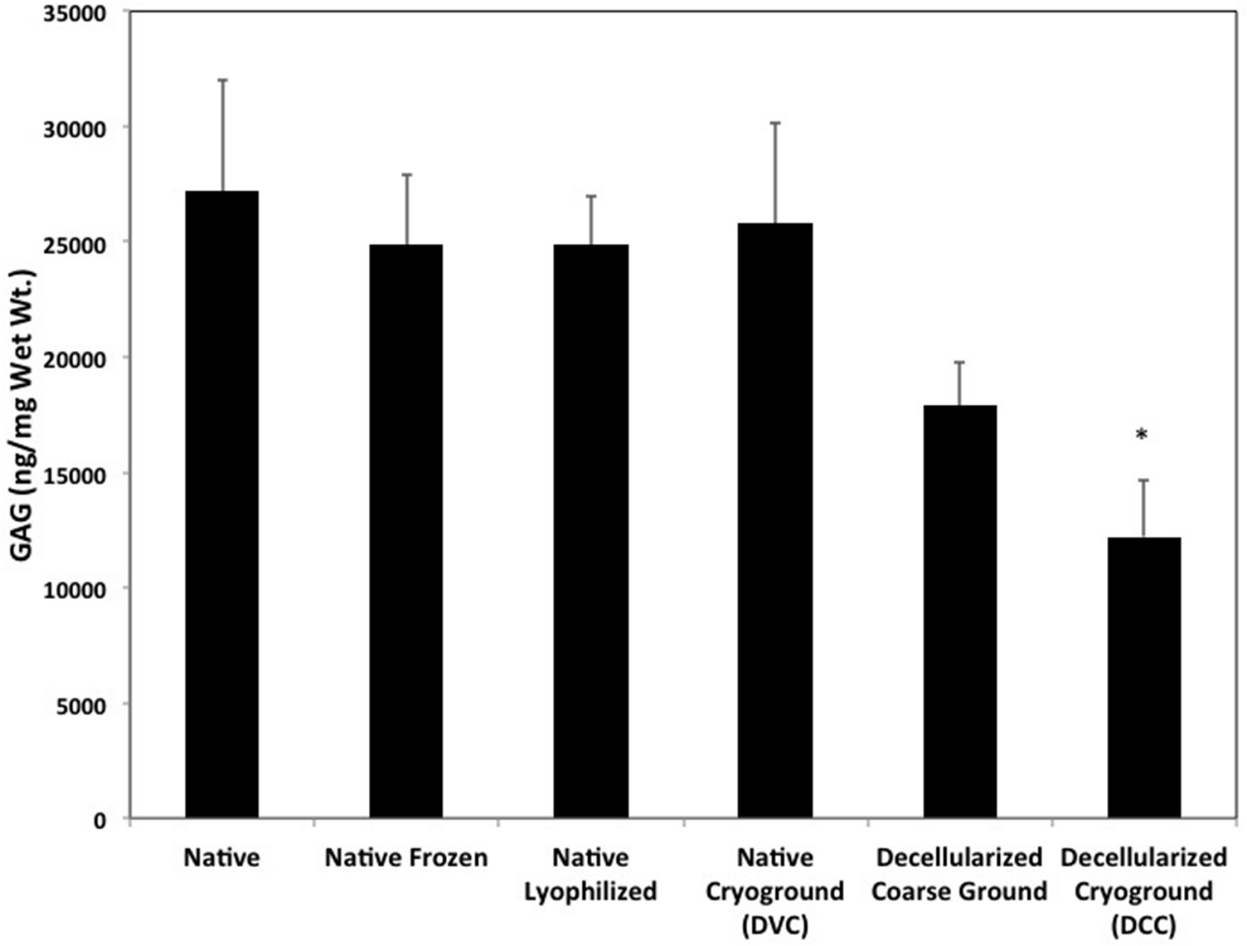
DMMB assay results depicting change in glycosaminoglycan (GAG) content of cartilage matrix during physical and chemical decellularization. Only DCC (both physical and chemical methods) significantly reduced the GAG content in the cartilage matrix by 55%. * denotes significance (p<0.01) from native cartilage (n = 6). All results are reported as mean ± standard deviation.

**Fig 4:**
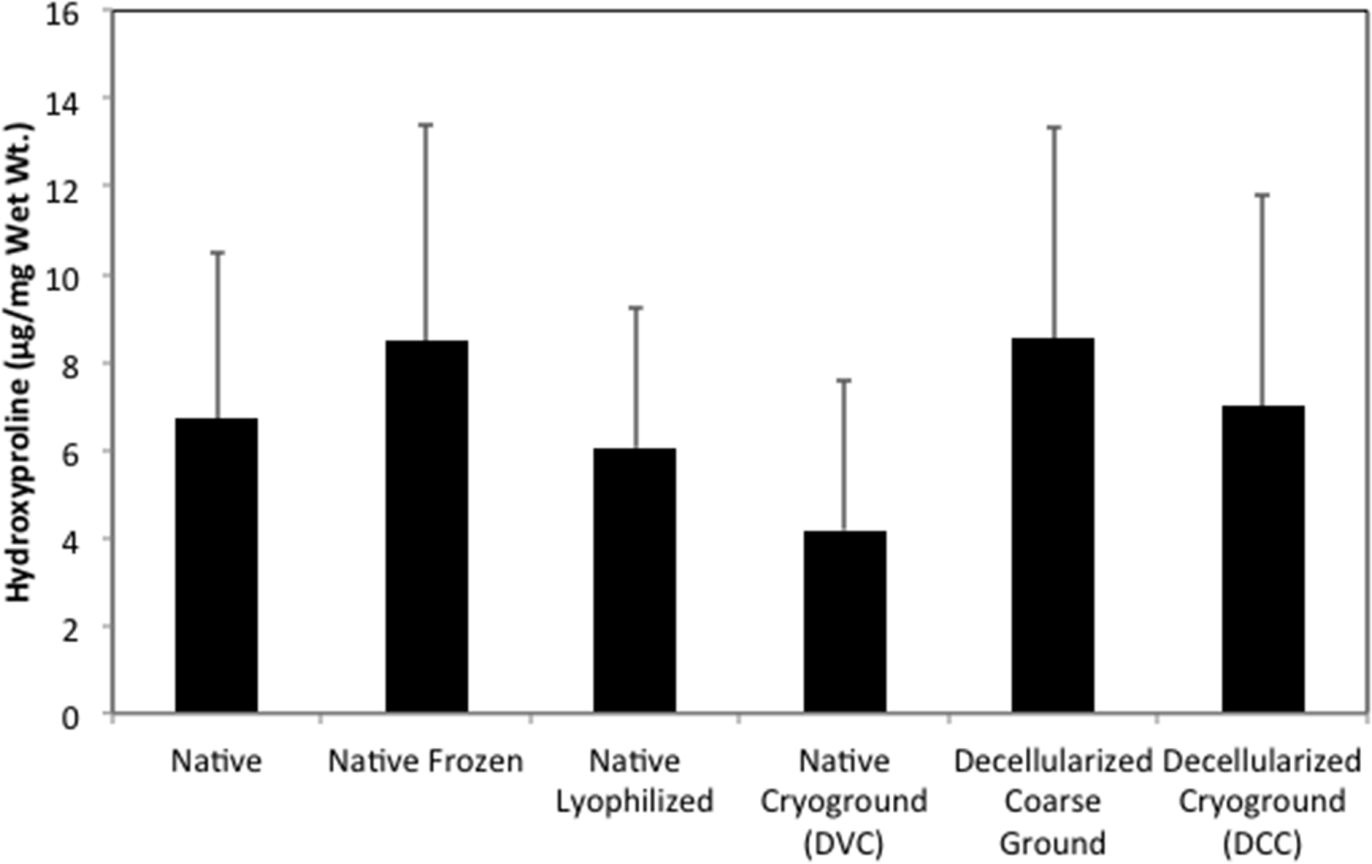
Hydroxyproline content of cartilage matrix during physical and chemical decellularization process. No statically significant differences were observed during the processing (n = 6). All results are reported as mean ± standard deviation.

Following coarse grinding, chemical decellularization, and cryo-grinding there was an 86% reduction in DNA content (p<0.01) (Fig 2) and a 55% reduction in GAG content (p<0.01) (Fig 3). However, there was no significant difference in hydroxyproline content during any steps of the tissue processing (Fig 4). Freezing, lyophilization, and cryo-grinding had no significant effect on DNA or GAG content in the tissue.

### SEM Imaging

**Fig 5:**
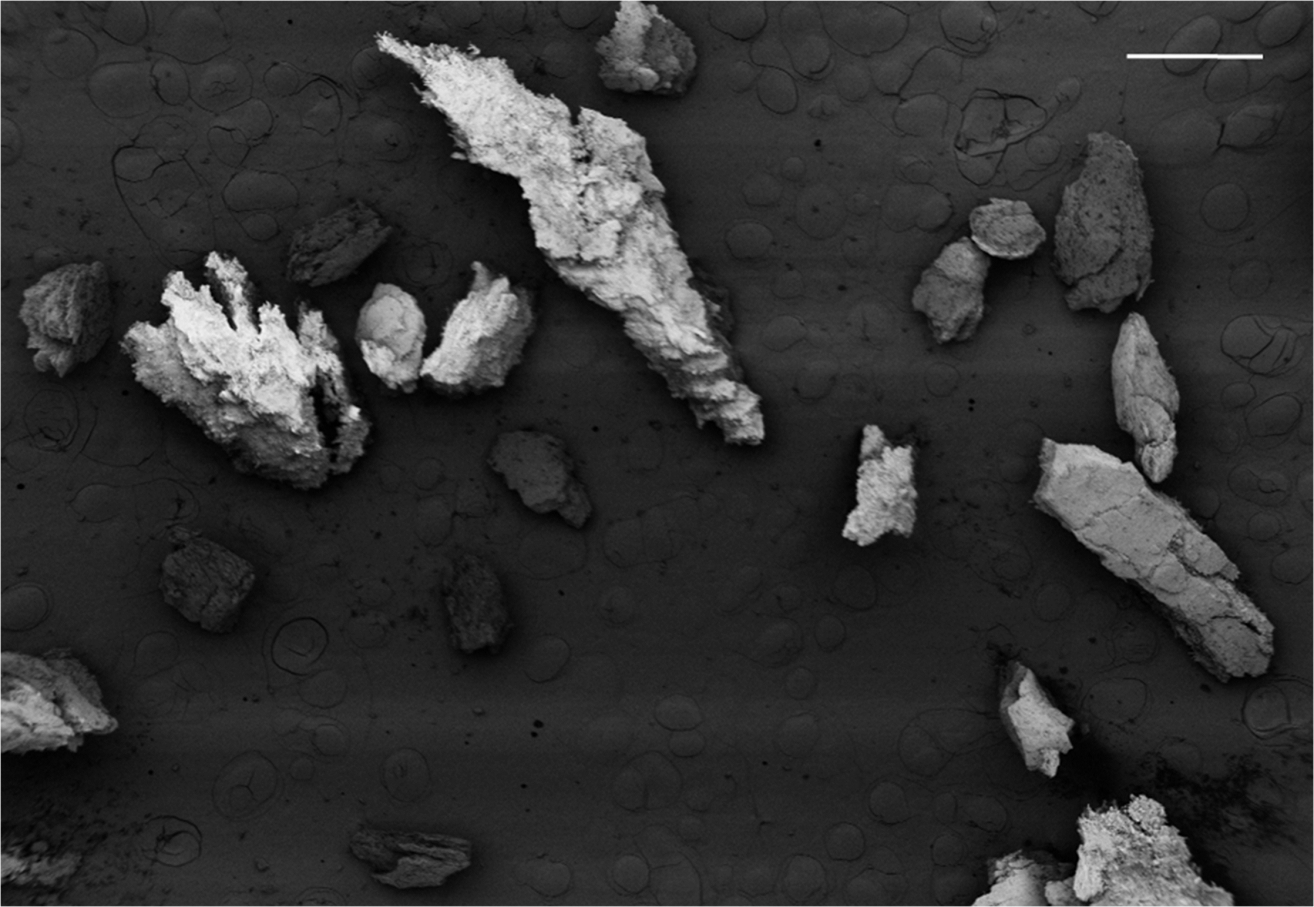
SEM Image of Cryo-ground DCC. The scale bar is 1 mm.

SEM imaging revealed that DCC particles are heterogeneous in morphology and size (Fig 5).

### Cell Viability

**Fig 6:**
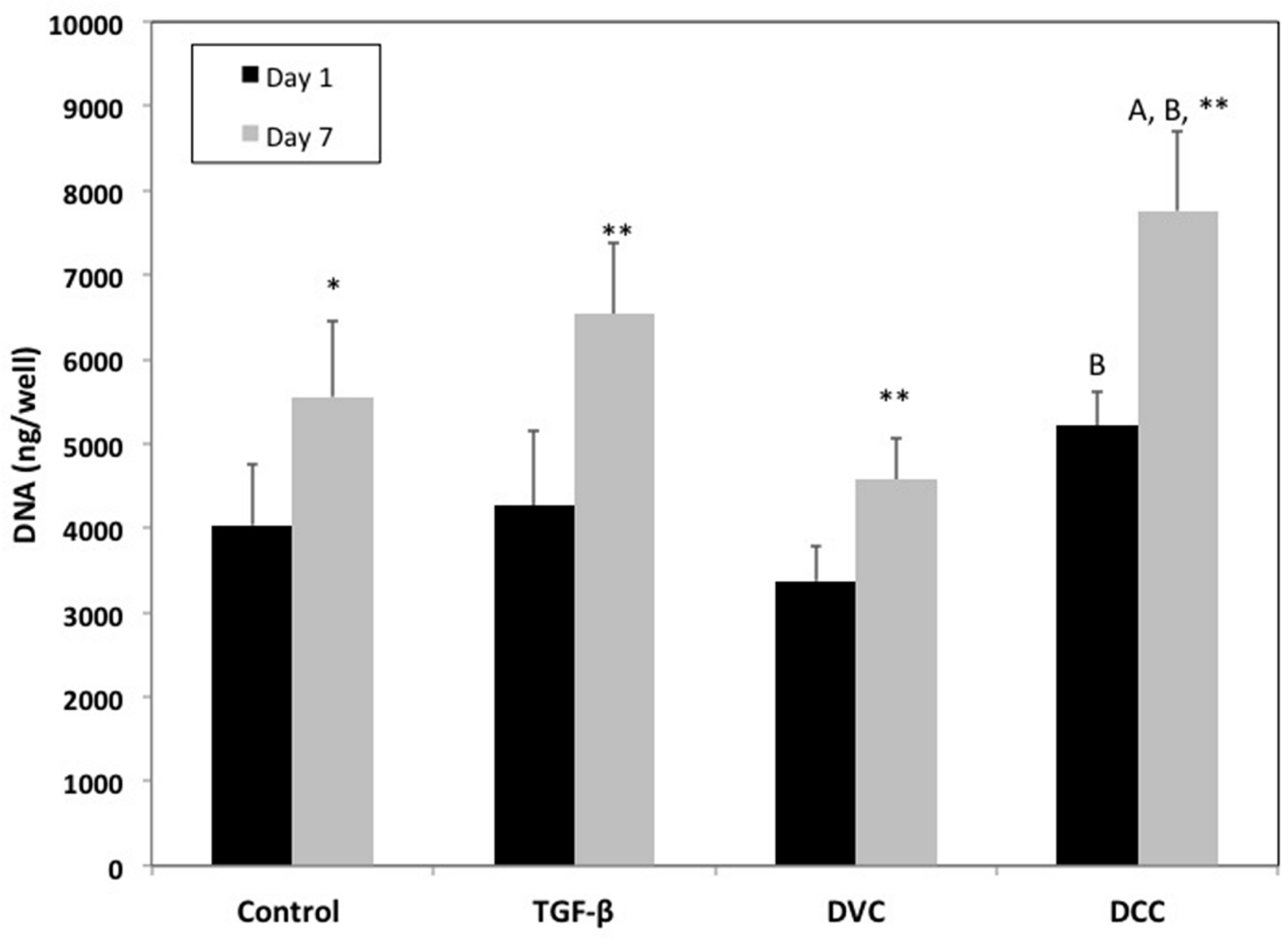
DNA content of cell pellets at days 1 and 7 (n = 5). All groups significantly increased DNA content between 1 and 7 days. * p<0.05 between day 1 and 7, ** p<0.01 between day 1 and 7, A = p<0.05 between DCC and control, and B = p<0.01 between DCC and DVC. All results are reported as mean ± standard deviation.

DNA was quantified in cell pellets at 0 and 7 days to determine cell proliferation on devitalized and decellularized cartilage. All cell pellets showed a significant increase in DNA amount over 7 days (p<0.05) (Fig 6). At 7 days, cell pellets with DCC had approximately 40% more DNA than the DVC cell pellets at the same time (p<0.01) (Fig 6). DCC pellets also had approximately 30% more DNA than the negative control group (p<0.01) (Fig 6).

### Gene Expression

**Fig 7:**
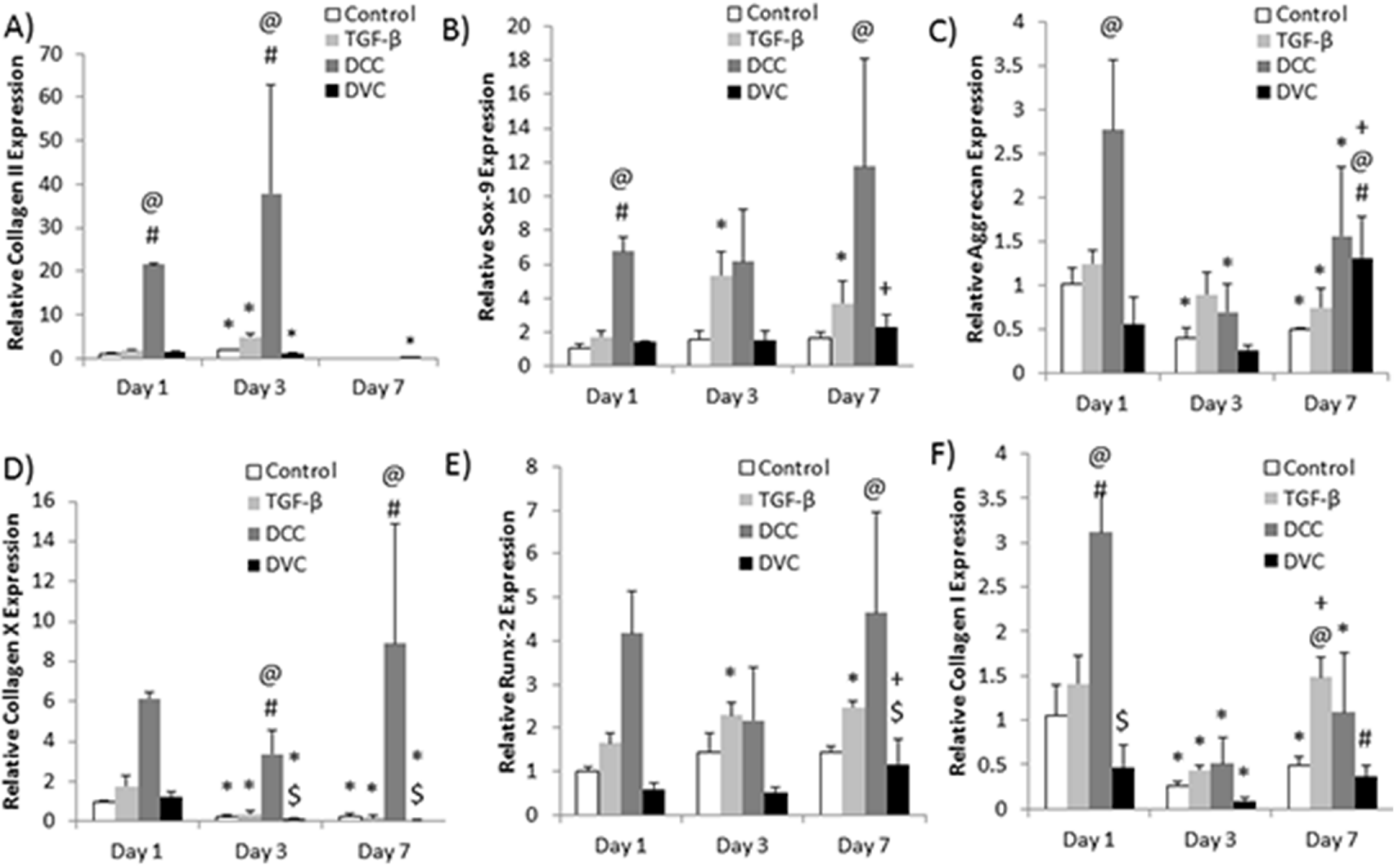
Relative expression of chondrogenic and osteogenic gene markers (n = 5). A) collagen II, B) Sox-9, C) aggrecan, D) collagen X, E) Runx-2, and F) collagen I. @ denotes significant difference from control group at same time point, # denotes significant difference from TGF-β group at same time point, * denotes significant difference between day 1 value, + denotes significant difference from previous time point, $ denotes significant difference from DCC group at same time point.

qPCR was used to determine the expression of both chondrogenic and osteogenic genes. DCC pellet group gene expression showed over a 90% increase in collagen II expression compared to both the TGF-β and negative control groups at day 1 (p<0.01) (Fig 7). Collagen II expression remained greater than both the TGF-β and negative control groups at day 3 (p<0.01). The DCC pellet group also showed 75% greater upregulation of Sox9 compared to the TGF-β group at day 1 (p<0.01) (Fig 7). A 60% increase in aggrecan expression was observed in the DCC group compared to the negative control at day 1 (p<0.01) (Fig 7). The osteogenic marker collagen X was expressed 5 times greater in the DCC group compared to the negative control group at day 7 (p<0.01) (Fig 7). Collagen X was also expressed 45 times greater in DCC than in the TGF-β group (p<0.01). Runx2 expression in the DCC group was also upregulated compared to the negative control group by 75% at day 7 (p<0.05) (Fig 7). Collagen I expression in the DCC group was also over 50% greater than both the negative control and TGF-β groups at day 1 (p<0.01), but significantly decreased at both days 3 and 7 compared to day 1 (p<0.05) (Fig 7).

The DVC group saw no significant difference in collagen II expression from both the negative control and TGF-β groups at days 1 and 3 (Fig 7). Aggrecan expression in the DVC group was over 40% greater than both the TGF-β and negative control groups at day 7 (p<0.05), however, it was not statistically significant from the DCC group at the same time (Fig 7). The DVC group Sox9 expression increased approximately 30% between days 3 and 7 (p<0.05) but was not significantly greater than either control group or the DCC group (Fig 7). Expression of osteogenic marker collagen X was 100% less in the DVC group compared to the DCC group at day 7 (p<0.01) (Fig 7). Runx2 expression was also 30% less in the DVC group compared to the DCC group at day 7 (p<0.01) (Fig 7). Collagen I expression in the DVC group was 60% less than the DCC group at day 1 (p<0.01) and 3 times less than the TGF-β group at day 7 (p<0.05) (Fig 7).

## Discussion

Synthetic biomaterials have achieved clinically relevant mechanical performance for osteochondral implantation; however, they have not been successful at fully regenerating functional articular cartilage. The use of ECM-based materials for osteochondral tissue engineering is a promising avenue because of the ECM materials’ ability to mimic the native cartilage environment by providing cells with adhesion sites and biochemical signals that aid in recruiting and differentiating stem cells for tissue regeneration. ECM-based materials may be able to provide these signals to cells without manipulation of the material with added biological factors (e.g., growth factors or adhesion peptides).

Successful decellularization of articular cartilage has previously been accomplished using different methods with differing results with respect to the remaining biochemical content, cell removal, and mechanical performance [9, 19, 29, 30]. Cartilage ECM has also been used as a scaffolding material that has only been physically devitalized as opposed to being decellularized. The different effects that devitalization vs. decellularization have on the cartilage matrix until now have not been fully characterized throughout each respective process. Moreover, DVC and DCC have not been directly compared *in vitro* or to a positive control such as TGF-β. The current study has shown that DCC may outperform both DVC and TGF-β at inducing chondrogensis in BMSCs *in vitro*. However, a limitation of this study is the inclusion of FBS in all of the experimental groups except the TGF-β supplemented group. Although we cannot conclude why it appears that DCC may outperform DVC in its chondrogenic potential, it might be possible that by removing cellular content and some GAGs allows cells to better infiltrate and attach to the DCC matrix rather than the DVC matrix.

The current combined physical and chemical decellularization method was successful at reducing the amount of detectable dsDNA within the cartilage matrix by 86% (p<0.01). Freezing, lyophilization, and cryogrinding, all common devitalization techniques, as expected were not effective at removing any dsDNA from the matrix. Although the DNA content of the DCC was significantly reduced, the GAG content was also significantly reduced by 55% (Fig 3). Retaining GAG content in cryo-ground DCC is crucial as the size of cryo-ground DCC versus coarse ground DCC is more ideal to incorporate into 3D scaffolds, such as pastes, hydrogels, and microspheres.

Currently, little is understood about the mechanism by which cartilage matrix materials induce chondrogenesis *in vitro*. Retention of GAG within the matrix may be beneficial for chondroinduction based on previous studies citing that GAGs such as chondroitin sulfate and aggrecan may have chondroinductive effects *in vitro* [25, 31, 32]. Although GAG retention may be beneficial for cell signaling, a partial reduction in GAG content may be beneficial to create a less dense matrix that allows for cell infiltration and migration [13].

In the current study, BMSCs cultured with DCC showed increased expression of both chondrogenic and osteogenic gene markers compared to the negative control group. Even though some evidence of osteogenesis (e.g., significant upregulation of Runx-2 and Collagen X genes, which are indicative of hypertrophic chondrocytes) was observed alongside chondrogenesis in this *in vitro* experiment, it is also hypothesized that in future work where this material is implanted in an osteochondral defect, for example, that the complex environment of mechanical and chemical signaling *in vivo* may possibly lead to regional differentiation, from chondrogenesis at the surface to possibly endochondral ossification below. Additionally, it should also be emphasized that this study only observed gene expression changes over a one week period. Now that initial gene expression changes are known, there is a motivation for future work to observe long term effects of exposure to cartilage matrix. Within one week however, the DVC group showed lower expression of the osteogenic markers collagen X and Runx2 than the DCC group. Although the DVC seemed to limit osteoinduction in the BMSCs, the chondrogenic gene markers collagen II, aggrecan, and Sox9 were significantly upregulated in the DCC group compared to the DVC group. This suggests that chondroinduction by DCC is not affected by the decellularization method. Additionally, comparison between chondroinduction via TGF-β and DCC showed that DCC chondroinduction was not statistically significant with respect to expression of aggrecan and Runx2 at all time points. However, chondroinductive markers including collagen II and Sox9 were expressed 20 and 4 times higher respectively in the DCC group compared to the TGF-β group at day 1. Similar expression of collagen I between the DCC group and the TGF-β group was also seen at 3 and 7 days. Chondroinductive effects appeared to be strongest in terms of collagen II expression at earlier time points, and it is unclear why the collagen II expression decreased at day 7. Possibilities may include a temporary pause in collagen II synthesis, a transition to higher metabolic activity in lieu of matrix synthesis, and/or a phenotype shift. Future investigations into underlying mechanisms will be of value to better elucidate temporal variations in gene expression levels.

Overall, these results suggest that DCC may outperform TGF-β at inducing chondrogenesis but does not confirm that latent TGF-β within the DCC matrix is responsible for the observed chondrogenesis. Future work will certainly need to explore whether there are latent and bioactive growth factors within the cartilage matrix and to determine whether they play a role in inducing chondrogenesis. Additionally, because in this work only one concentration of DCC was explored, it would also be of interest to study the role of the concentration of DCC on chondrogenesis in future studies.

It is still unclear whether full decellularization of articular cartilage is necessary when delivering cartilage matrix materials to osteochondral defects *in vivo*. Decellularization may reduce the antigenicity of the matrix by removing cellular materials that have been previously shown to elicit immune responses such as human leukocyte antigens (HLA) and the alpha-Gal epitope. It is also important to emphasize that in this study, we obtained porcine cartilage matrix and cultured it with rat BMSCs. It would be of interest to compare the same species for both the cell source and cartilage matrix. Although using the same cartilage species would certainly be ideal to limit an adverse immunological response, it would also be possible to use porcine (or other xenograftic tissue) as long as there was successful removal of the alpha-Gal epitope. Previous studies have shown successful removal of the alpha-Gal epitope through chemical decellularization but not physical devitalization alone [13]. Further investigations are needed to evaluate immunogenicity and successful removal of the alpha-Gal epitope. Additionally, future studies can explore how the decellularization method affects the overall chondrogenic potential of the cartilage ECM.

Although not specifically explored in the current study, a delivery method of this material must also be considered to create a tissue engineering scaffold with that contains both the benefits of cartilage matrix materials with enhanced mechanical performance. The development of an acellular, non-biologically modified biomaterial that has the ability to induce chondrogenesis is of particular importance to the tissue engineering field because of it may have the ability to replace current surgical techniques with more positive outcomes. The ECM material approach is also highly attractive from both a regulatory and commercialization standpoint because of the cost of materials and no added biologic factors.

This is the first study to fully characterize both DCC and DVC through the respective decellularization and devitalization processes. Addtionally, this is the first study to directly compare the bioactivity of non-crosslinked DCC, DVC, and TGF-β *in vitro*. DCC was found to have superior effects compared to both DVC and TGF-β at inducing chondrogensis and supporting cell proliferation. Athough there is still much to explore regarding the chondroinductive potential of DCC, the ability to influence cell differentiation without additional biological manipulation makes DCC a promising biomaterial for use in future cartilage tissue engineering applications.
